# Cognitive Outcomes in Children With Conditions Affecting the Small Intestine: A Systematic Review and Meta-analysis

**DOI:** 10.1097/MPG.0000000000003368

**Published:** 2021-12-15

**Authors:** Lotte E. Vlug, Merel W. Verloop, Bram Dierckx, Lotte Bosman, Jurgen C. de Graaff, Edmond H.H.M. Rings, René M.H. Wijnen, Barbara A.E. de Koning, Jeroen S. Legerstee

**Affiliations:** ∗ Division of Gastroenterology, Department of Pediatrics; † Department of Child and Adolescent Psychiatry/Psychology; ‡ Department of Anesthesiology, Erasmus MC Sophia Children's Hospital, University Medical Center Rotterdam, Rotterdam; § Division of Gastroenterology, Department of Pediatrics, Willem Alexander Children's Hospital, Leiden University Medical Center, Leiden; || Department of Pediatric Surgery, Erasmus MC Sophia Children's Hospital, University Medical Center Rotterdam, Rotterdam, The Netherlands.

**Keywords:** cognition, intestinal failure, necrotizing enterocolitis, neurodevelopment, short bowel syndrome

## Abstract

**Objectives:**

The aim of the study was to assess cognitive outcomes in children with intestinal failure (IF) and children at high risk of IF with conditions affecting the small intestine requiring parenteral nutrition.

**Methods:**

EMBASE, Cochrane, Web of Science, Google Scholar, MEDLINE, and PsycINFO were searched from inception to October 2020. Studies were included constituting original data on developmental quotient (DQ), intelligence quotient (IQ) and/or severe developmental delay/disability (SDD) rates assessed with standardized tests. We used appropriate standardized tools to extract data and assess study quality. We performed random effects meta-analyses to estimate pooled means of DQ/IQ and pooled SDD rates (general population mean for DQ/IQ: 100, for percentage with SDD: 1.8%) for 4 groups: IF, surgical necrotizing enterocolitis (NEC), abdominal wall defects (AWD), and midgut malformations (MM). Associations of patient characteristics with DQ/IQ were evaluated with meta-regressions.

**Results:**

Thirty studies met the inclusion criteria. The pooled mean DQ/IQ for IF, NEC, AWD, and MM were 86.8, 83.3, 96.6, and 99.5, respectively. The pooled SDD rates for IF, NEC, AWD and MM were 28.6%, 32.8%, 8.5%, and 3.7%, respectively. Meta-regressions indicated that lower gestational age, longer hospital stay, and higher number of surgeries but not parenteral nutrition duration, were associated with lower DQ/IQ.

**Conclusions:**

Adverse developmental outcomes are common in children with IF and NEC, and to a much lesser extent in children with AWD and MM. It is important to monitor cognitive development in children with conditions affecting the small intestine and to explore avenues for prevention and remediation.

**An infographic is available for this article at:**.

**What Is Known/What Is New**

**What Is Known**

- Children with intestinal failure are at risk of delayed psychomotor and cognitive development.
- Early causes of intestinal failure include surgical necrotizing enterocolitis, abdominal wall defects, and midgut malformations. It is unclear whether neurodevelopmental delay seen in intestinal failure results from underlying disease or parenteral nutrition.

**What Is New**

- Adverse cognitive outcomes are common in children with intestinal failure and necrotizing enterocolitis but less in children with abdominal wall defects and midgut malformations.
- Those with a low gestational age, long hospitalization, multiple surgical procedures, but not necessarily long duration of parenteral nutrition-dependency, are especially prone.

In infants with conditions affecting the small intestine, the gut insufficiently absorbs nutrients and fluids needed for growth. Therefore, these infants depend on parenteral nutrition (PN) (1). Some of them (23%–35% of infants with surgically treated necrotizing enterocolitis (NEC) (2), 10% to 34% of infants with abdominal wall defects (3,4), 12% of infants with intestinal atresia (5), around 80% of children with pediatric intestinal pseudo-obstruction syndrome (6) and almost all children with microvillus inclusion disease (7)) become long-term PN-dependent and therewith develop intestinal failure (IF). New challenges in children with IF have become apparent, including neurodevelopment. Hukkinen et al reviewed available literature and concluded that children with IF are at significant risk of delayed psychomotor and cognitive development but this was based on few and small studies with varying methodology (8). It is unclear if the neurodevelopmental deficits are related to the prolonged administration of PN or to other disease-specific or more generic factors. Systematically evaluating available literature concerning cognitive development in children with neonatal underlying diseases of IF will enhance our knowledge on early protective and risk factors for less optimal outcomes in children with IF. This will help clinicians to better inform parents and to take measures that support vulnerable children to prevent or remediate deficits later in life.

The aims of this systematic review and meta-analysis were to assess cognitive outcomes both in children with IF receiving long-term PN and in children at high risk of developing IF, and to examine the influence of patient characteristics on reported outcomes.

## METHODS

The protocol and objectives for this study were established a priori and registered in PROSPERO, an international database of prospectively registered systematic reviews in health and social care (protocol number 173400). The systematic review and meta-analysis were performed according to the guidelines of the Preferred Reporting Items for Systematic Reviews and Meta-Analyses (PRISMA) statement (9).

### Search Strategy

A systematic literature search was conducted on October 26, 2020 in EMBASE, Cochrane, Web of Science, Google Scholar, MEDLINE, and PsycINFO by a biomedical information specialist of the Medical Library of the Erasmus University Medical Center. The inclusion criteria were studies reporting on cognitive outcomes in children with IF and at high risk of IF, with no limitation on publication date (to include as much relevant data as possible on these rare diseases as age of study is not an important differentiating factor). The following search terms were used: neonate, infant, child, adolescent; neurodevelopment, cognition, learning disorder, intelligence quotient (IQ); IF, PN, and the different underlying diseases of IF as described in File S1 (Supplemental Digital Content 1). Only studies using standardized developmental/intelligence tests and/or a clear definition of severe developmental delay/disability (SDD) based on cognitive testing were included. These tests include the Bayley Scales of Infant Development (BSID) (without motor functioning scale), the Mullen Scales of Early Learning (MSEL) (without motor functioning scale), the Wechsler Preschool and Primary Scale of Intelligence (WPPSI), and the Wechsler Intelligence Scale for Children (WISC), which are all standardized validated tools. Studies were excluded if they were not written in English, not in human subjects, and if they were reviews, case reports or case series including less than 10 patients. Abstracts, posters, editorials, letters, and books were also excluded.

### Study Selection and Data Extraction

Two investigators (L.E.V. and M.W.V.) independently screened all titles and abstracts in EndNote, blinded to each other's decisions. A selection was made for full-text screening based on title and abstract, after which full-text assessment led to final inclusion. The reference lists of the included studies and reviews were examined for additional eligible studies. In case of discrepancy at any stage, the reviewers tried to reach consensus by discussion and if not reached, a third independent reviewer was consulted (J.S.L.). If studies were based on an identical cohort sample, only 1 study was included (the most recent study with the biggest sample size).

The following data were extracted into Comprehensive Meta-analysis software version 2.0 (Biostat Inc, Englewood, NJ): study design and setting, patient characteristics (number of patients, sex, gestational age, birth weight, underlying disease, number of surgeries, duration of hospital admission, PN-dependency duration, age at cognitive assessment), study objective, intelligence test, mean developmental quotient (DQ) (assessed with BSID or MSEL) and IQ (assessed with WPPSI or WISC), and number of patients with SDD (defined as a DQ/IQ of >2 standard deviations [SDs] below the population mean; this was a DQ/IQ of <70 since in the general population the mean DQ/IQ is 100 and SD is 15). DQ was equivalent to mental development index for BSID-II, cognitive composite score for BSID-III and early learning composite for MSEL; IQ was equivalent to full scale IQ for WPPSI and total IQ for WISC (10). In case of missing data in a specific study, the corresponding authors were contacted by email and asked to provide us with missing information (eg, means and SDs for PN-dependency duration).

### Quality Assessment

The quality of the individual studies was assessed using checklists from the National Heart, Lung, and Blood Institute (NIH Quality Assessment Tools for Observational Cohort and Cross-Sectional studies, and for Case-Control Studies) (11). Criteria assessing internal validity and risk of bias were checked for every study and the quality of each study was rated independently by 2 authors (L.E.V. and M.W.V.) as “Good,” “Fair,” or “Poor.” In case of disagreement between the authors, consensus was reached through discussion or by consulting a third author (J.S.L.). The items used for quality assessment are shown in Table S1 (Supplemental Digital Content 2).

### Statistical Analysis

Descriptive statistics are reported as frequency (percentage) for categorical variables and mean (SD) for continuous variables. When medians and interquartile ranges or ranges were given, means and SDs were estimated using Wan's and Hozo's method in order to combine results for the meta-analysis (12,13). Because of expected between-study heterogeneity because of varying underlying diseases and age ranges, we performed random effects meta-analyses to calculate pooled means of DQ/IQ with 95% confidence intervals (CIs), and the pooled prevalence of SDD with 95% CIs. Inverse variance weighting was conducted according to the number of patients included. Data were analyzed separately for subgroups of patients: IF and short bowel syndrome, surgical NEC and intestinal perforation, abdominal wall defects (gastroschisis and omphalocele), and midgut malformations (intestinal atresia, intestinal stenosis, or intestinal malrotation). We also performed subgroup analyses for children aged <3 years (assessing DQ) and older children (assessing IQ). Pooled estimates were visualized in forest plots, in which DQ/IQ and percentages of patients with SDD were compared with the general population mean. For DQ/IQ, this was a general population mean of 100; for SDD, we used a mean percentage of 1.8%, known from a population-based meta-analysis (14). Heterogeneity was assessed using Cochran Q homogeneity and I^2^-statistic (percentage of unexplained variance) for the degree of inconsistency. Values of I^2^ of ≥75% indicate substantial heterogeneity (15). Publication bias was examined in a funnel plot and with Egger tests (16). Meta-regressions were performed to examine the impact of the moderator variables’ duration of PN-dependency, age at time of cognitive assessment, gestational age, duration of hospital stay, and number of surgeries on DQ/IQ. Statistical analyses were performed using Comprehensive Meta-analysis software version 2.0 (Biostat Inc, Englewood, NJ) and the *meta*(17) and *metafor*(18) packages from R version 4.0.3 (R Foundation for Statistical Computing, Vienna, Austria, *http://www.R-project.org/*).

## RESULTS

### Study Selection

**FIGURE 1:**
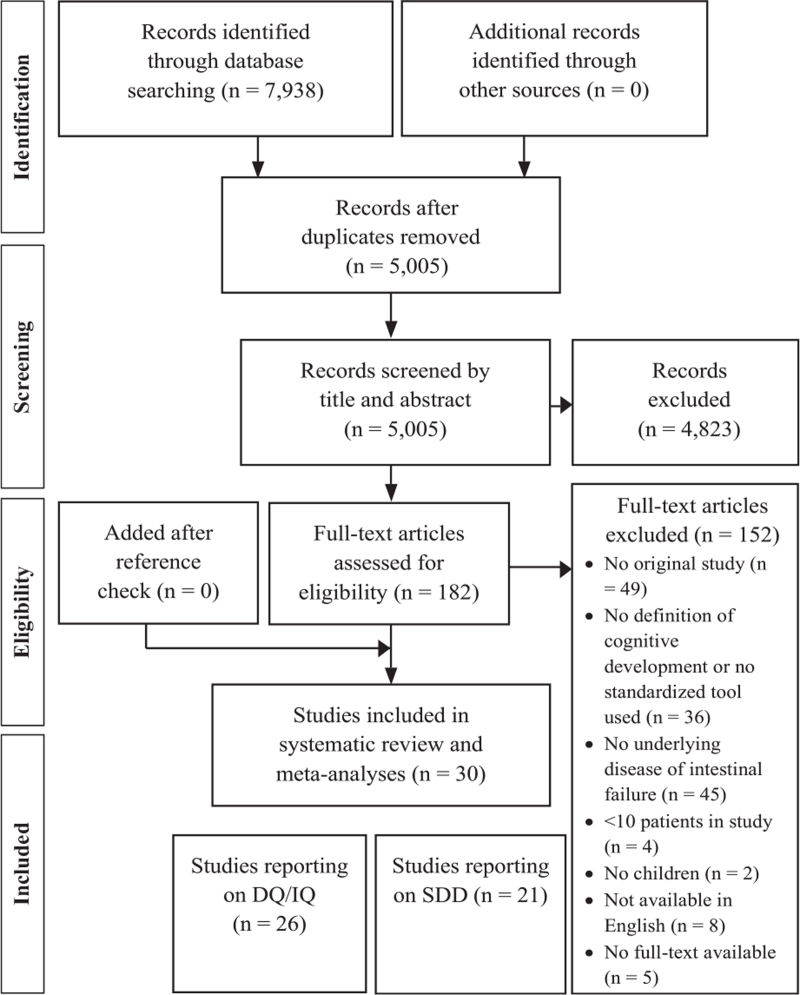
Flow chart of study inclusion in the systematic review and meta-analysis. DQ = developmental quotient; IQ = intelligence quotient; SDD = severe developmental delay/disability.

The study selection process is displayed in Figure 1. Following title and abstract screening, 182 out of 5005 studies were eligible (98% reviewer consensus). Full-text screening led to inclusion of 33 studies (86% reviewer consensus). The corresponding authors of 4 studies were able to provide us with additional data (19–22). After taking into account sample overlap, 30 articles were selected for data extraction. Twenty-six studies were included in the meta-analysis assessing DQ/IQ and 21 studies in the meta-analysis assessing prevalence of SDD.

### Study Characteristics

Eleven studies were of retrospective design (19,23–32), 19 of prospective design (20–22,33–48). Sample size across studies ranged from 10 to 449 patients. Children were assessed for cognitive development at an age ranging between 1 and 16 years old. In most studies (66%), the BSID was used, at around 2 years of age. Six studies involved children with chronic IF/short bowel syndrome, 12 with surgical NEC/intestinal perforation, 10 with abdominal wall defects, and 2 with midgut malformations. No studies were found involving children with enteropathies or motility disorders (except the studies on chronic IF but not as separate underlying disease). In Table 1, the study characteristics of the included studies are shown.

**TABLE 1:** Study characteristics

Number	Study	Year	Inclusion period	Sample size, N	Boys, n (%)	Underlying disease	Duration of PN dependency, weeks mean (SD)	Gestatio-nal age, weeks mean (SD)	Age at follow-up, years mean (SD) or range	Assessment tool	DQ/IQ mean (SD)	Severe developmental delay/disability, *n* (%)	Quality assessment score
Chronic intestinal failure/short bowel syndrome													
1	Chesley et al	2016	Unknown	15	12 (80)	Surgical and functional^†^	123.0 (68.7)	34.8 (4.2)	2.4 (1.3)	BSID-II WPPSI-II	79.5 (15.6)	3 (20)	Fair
2	Gold et al	2020	2012 to 2016	28	15 (54)	Surgical and functional^†^	51.6 (28.4)	35.5 (5.5)	6.4 (0.8)	WPPSI-IV WISC-IV/V	89.0 (17.2)	NA	Fair
3	Gunnar et al	2020	2017 to 2018	30/26^∗^	24 (80)	Surgical and functional^†^	56.5 (100.6)	35.0 (7.8)	7.5 (4.1)	WPPSI-III WISC-IV	78 (20.4)	10 (35)	Good
4	O’Connor et al	1988	1977 to 1986	12	9 (75)	Surgical and functional^†^	227.2 (67.6)	NA	4.2 to 7.8	WPPSI WISC-R	101.5 (14.3)	NA	Fair
5	So et al	2019	2011 to 2013	31/30^∗^	17 (55)	Surgical and functional^†^	31.3 (64.6)	34 (4.7)	2.2 to 2.7	MSEL	81.0 (19.4)	8 (27)	Good
6	Sorrell et al	2017	2011 to 2013	13	6 (46)	Surgical and functional^†^	27.8 (20.0)	27.9 (3.8)	1.0	BSID-III	74.3 (15.9)	NA	Fair
Surgical necrotizing enterocolitis/intestinal perforation													
7	Adesanya et al	2005	1996 to 1999	28/21^∗^	22 (79)	Surgical NEC with intestinal perforation	14.9 (6.1)	26 (2)	1.0	BSID-II	77.0 (17.0)	7 (33)	Good
8	Allendorf et al	2018	2006 to 2013	24	NA	Surgical NEC	7.1 (4.5)	27.9 (4.4)	2.0	BSID-II	85.0 (17.3)	2 (8)	Fair
9	Fullerton et al	2018	1999 to 2012	449	260 (58)	Surgical NEC	NA	25.0 (2.0)	1.7 (0.1)	BSID-II BSID-III	NA	86 (19)	Fair
10	Hintz et al	2005	1995 to 1998	124/118^∗^	64 (52)	Surgical NEC	NA	NA	1.6 to 1.8	BSID-II	72.0 (18.0)	52 (44)	Fair
11	Humberg et al	2020	2009 to 2014	43	18 (42)	Surgical NEC	NA	26.1 (1.2)	5.0 to 6.0	WPPSI-III	85.0 (17.0)	NA	Fair
12	Kuik et al	2020	2010 to 2017	13	6 (47)	Surgical NEC	4.7 (3.8)	27.0 (1.1)	2.3 (0.4)	BSID-III	98.9 (15.3)	1 (8)	Fair
13	Martin et al	2010	2002 to 2004	42	NA	Surgical NEC	NA	NA	2.0 to 2.3	BSID-II	NA	20 (48)	Fair
14	Shah et al	2012	1998 to 2009	121/32^∗^	62 (51)	Surgical NEC	7.0 (5.4)	25.7 (2.1)	1.5 to 1.8	BSID-II	78.1 (3.9)	NA	Fair
15	Sung et al	2019	2013 to 2016	26	17 (65)	Surgical NEC	NA	25.8 (2.3)	1.5 to 2.0	BSID-II BSID-III	77 (24)	13 (50)	Fair
16	Ta et al	2011	1996 to 2002	19	12 (63)	Surgical NEC	5.1 (2.6)	31.8 (4.3)	9.8 (2.0)	WISC-III	86.5 (13.1)	7 (37)	Fair
17	Wadhawan et al	2014	2000 to 2005	472/174^∗^	275 (58)	Surgical NEC	8.2 (5.2)	25.6 (1.9)	1.6 to 1.8	BSID-II	NA	88 (51)	Good
18	Zozaya et al	2020	2010 to 2011	61/22^∗^	44 (72)	Surgical NEC	NA	25.4 (1.7)	1.5 to 2.5	BSID-III	91 (4.8)	6 (27)	Fair
Abdominal wall defects													
19	Bevilacqua et al^‡^	2015	2008 to 2012	18	10 (56)	Gastroschisis, omphalocele	NA	33.3 (1.3)	1.0	BSID-III	102.3 (10.1)	NA	Good
20	Burnett et al	2018	2009 to 2014	59	29 (49)	Gastroschisis, omphalocele	NA	36.6 (1.7)	2.3 (0.3)	BSID-III	101.6 (14.2)	NA	Good
21	Danzer et al	2010	2002 to 2007	15	9 (60)	Giant omphalocele	3.0 (1.7)	35.5 (3.3)	1.0 (0.6)	BSID-II BSID-III	79.5 (19.1)	7 (47)	Fair
22	Ginn-Pease et al	1991	1972 to 1981	22	8 (36)	Gastroschisis, omphalocele	3.4 (3.3)	NA	10.1 (2.9)	WISC-R	100.1 (15.9)	NA	Fair
23	Harris et al	2016	1992 to 2005	39	17 (44)	Gastroschisis	NA	36.0 (2.3)	>5	WPPSI-III WISC-IV	98.2 (10.7)	0 (0)	Fair
24	Hijkoop et al^§^	2018, 2019	2000 to 2012	103/84^∗^	47 (46)	Gastroschisis, omphalocele	10.6 (9.6)	33.8 (1.2)	2.0	BSID BSID-II	95.7 (19.4)	6 (7)	Good
25	Lap et al	2017	1999 to 2006	16	9 (56)	Gastroschisis	15.8 (16.2)	37.1 (3.0)	8.8 (2.3)	WISC-III	92.3 (13.3)	1 (6)	Good
26	Sirichaipornsak et al	2011	2007 to 2008	15	11 (73)	Gastroschisis	6.0 (4.1)	36.6 (1.7)	1.8 (0.3)	BSID-III	95.0 (8.9)	0 (0)	Fair
27	South et al	2008	2003 to 2005	17	6 (35)	Gastroschisis	4.1 (2.8)	35.5 (1.9)	1.7 (0.3)	BSID-II	101.0 (19.0)	1 (6)	Fair
28	Van Manen et al	2013	2005 to 2008	61/39^∗^	38 (60)	Gastroschisis	6.1 (9.2)	36.2 (2.2)	2.1 (0.7)	BSID-III WPPSI-III	NA	0 (0)	Fair
Midgut malformations													
29	Bevilacqua et al^‡^	2015	2008 to 2012	34	18 (53)	Volvulus, intestinal atresia	NA	34 (1.5)	1.0	BSID-III	101.4 (9.4)	NA	Good
30	Elsinga et al	2013	1995 to 2002	27	13 (48)	Intestinal atresia/stenosis/malrotation	NA	36.1 (3.8)	9.5 (1.9)	short WISC-III	97.3 (16.4)	1 (4)	Good

Data are presented as n (%) or mean (SD). Severe developmental delay/disability was defined as deviation in DQ/IQ of >2 SD below the normal population mean (DQ/IQ < 70). BSID = Bayley Scales of Infant Development; DQ = developmental quotient; IQ = intelligence quotient; MSEL = Mullen Scales of Early Learning; NA = not assessed; PN = parenteral nutrition; SD = standard deviation; WISC = Wechsler Intelligence Scale for Children; WPPSI = Wechsler Preschool and Primary Scale of Intelligence.

∗ Cognitive development was evaluated in a selection of the study population.

† Surgical includes NEC, volvulus, meconium peritonitis, intestinal atresia, gastroschisis; functional includes dismotility, enteropathy.

‡ In the same article, 2 underlying disease groups were evaluated and therefore shown separately.

§ In 2 articles, the same underlying disease group (abdominal wall defects separated in gastroschisis and omphalocele) from the same cohort and time period was evaluated and therefore shown combined.

### Quality Assessment

Ten studies had an overall rating of “Good.” 20 studies were rated “Fair,” and none were rated “Poor.” In general, studies lacked sample size justification and adjustment for key potential confounding variables. The quality rating per study is shown in Table 1.

### Meta-analyses

#### Developmental Quotient/Intelligence Quotient

**FIGURE 2:**
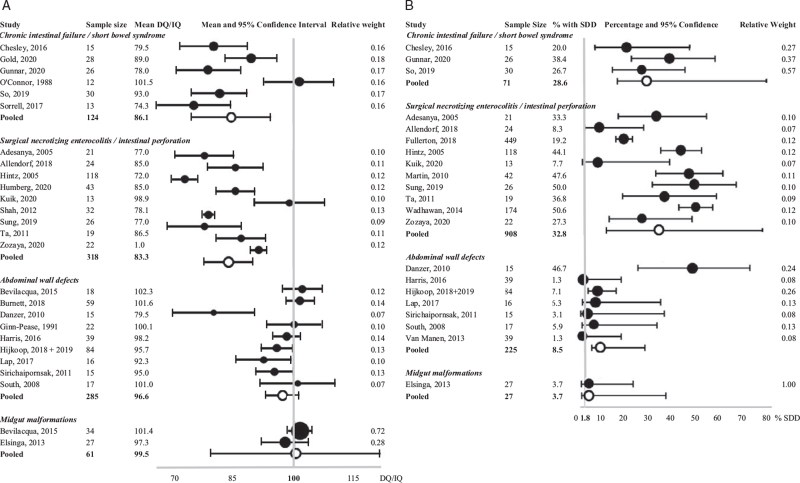
Forest plots of meta-analyses of the pooled developmental quotient/intelligence quotient (2A) and SDD (DQ/IQ < 70) rates (2B) divided in subgroups of underlying diseases, based on random effects analysis. DQ = developmental quotient; IQ = intelligence quotient; SDD = severe developmental delay/disability. The vertical grey line in (2A) represents the normal population mean of DQ/IQ (mean = 100, standard deviation = 15). The vertical grey line in (2B) represents the prevalence of SDD in children, known from a population-based meta-analysis (1.8%) (13).

The meta-analysis for pooled means of DQ/IQ included 788 patients from 26 nonoverlapping studies. The highest DQ/IQ were found in children with midgut malformations and abdominal wall defects (mean 99.5 (n = 2 studies, n = 61 patients; 95% CI 89.2–109.8) and 96.6 (n = 9 studies, n = 285 patients; 95% CI 91.6–101.6), respectively), followed by children with IF (mean 86.1 (n = 6 studies, n = 124 patients; 95% CI 79.7–92.5)), and the lowest scores were seen in children with surgical NEC/intestinal perforation with mean 83.3 (n = 9 studies, n = 318 patients; 95% CI 78.2–88.4). Estimates of DQ/IQ for each study are visualized in comparison with the general population mean in the forest plot from Figure 2A. When looking at children ages <3 years (assessed with BSID or MSEL) separately, pooled mean DQ in IF was 84.1 (n = 2 studies, n = 43 patients; 95% CI 70.8–97.4), 82.6 in surgical NEC/intestinal perforation (n = 7 studies, n = 256 patients; 95% CI 75.7–89.6), 96.3 in abdominal wall defects (n = 6 studies, n = 208 patients; 95% CI 88.8–103.7) and 101.4 in midgut malformations (n = 1 study, n = 34 patients; 95% CI 83.9–118.9). For older children (assessed with WPPSI or WISC), pooled mean IQ in IF was 89.4 (n = 3 studies, n = 66 patients; 95% CI 82–96.8), 85.7 in surgical NEC/intestinal perforation (n = 2 studies, n = 62 patients; 95% CI 77.4–94.1), 97 in abdominal wall defects (n = 3 studies, n = 77 patients; 95% CI 90.1–103.8), and 97.3 in midgut malformations (n = 1 study, n = 27 patients; 95% CI 85.2–109.4).

#### Severe Developmental Delay/Disability

The meta-analysis for pooled prevalences of SDD (DQ/IQ < 70) included 1231 patients from 21 nonoverlapping studies. The lowest percentages were found in studies including children with midgut malformations and abdominal wall defects (3.7% (n = 1 study, n = 27 patients) and 8.5% (n = 7 studies, n = 225 patients; 95% CI 3.7–18.5), respectively) and the highest rates in children with surgical NEC/intestinal perforation with 32.8% (n = 10 studies, n = 908 patients; 95% CI 22–45.9). In children with IF, overall SDD was found in 28.6% (n = 3 studies, n = 71 patients; 95% CI 12.5–52.9). The percentages of SDD and 95% CI for each study are shown in the forest plot from Figure 2B. The 95% CI for 3 out of 4 pooled SDD rates do not include the 1.8% SDD rate of the general population. When looking at children ages <3 years (assessed with BSID or MSEL) separately, pooled SDD rate in IF was 26.7% (n = 1 study, n = 30 patients, 95% CI 5.7–68.5), 32.2% in surgical NEC/intestinal perforation (n = 9 studies, n = 889 patients; 95% CI 20.7–46.5), and 13.2% in abdominal wall defects (n = 4 studies, n = 131 patients; 95% CI 4.8–31.2). For older children (assessed with WPPSI or WISC), pooled SDD rate in IF was 38.5% (n = 1 study, n = 26 patients; 95% CI 22.1–57.9), 36.8% in surgical NEC/intestinal perforation (n = 1 study, n = 19 patients; 95% CI 18.7–59.7), 3.6% in abdominal wall defects (n = 2 studies, n = 55 patients; 95% CI 0.7–16.2), and 3.7% in midgut malformations (n = 1 study, n = 27 patients; 95% CI 0.5–22.1).

### Publication Bias

Funnel plots for DQ/IQ and SDD prevalence showed asymmetry (see Figures S1 and S2, Supplemental Digital Content 3 and 4)), but Egger's regression asymmetry tests did not confirm the presence of a significant publication bias for DQ/IQ (*P* = 0.386) or SDD (*P* = 0.115).

### Heterogeneity

Substantial heterogeneity was found between studies within the same disease groups (IF/short bowel syndrome: I^2^ = 84.8%, surgical NEC/intestinal perforation: I^2^ = 93.3%, abdominal wall defects: I^2^ = 71.2%), except for the midgut malformation group (I^2^ = 25.3%). Causes of heterogeneity may be explained by differences in patient characteristics that were analyzed in the meta-regressions.

### Meta-regressions

Meta-regression outcomes of the associations between the moderator variables and overall DQ/IQ are shown in Table 2. Duration of PN-dependency was not associated with DQ/IQ, neither was age at assessment. A lower gestational age, longer hospital stay, and more surgical procedures were all significantly related to a lower overall DQ/IQ (shown in the scatterplots of Figures S3–S5, Supplemental Digital Content 5, 6 and 7).

**TABLE 2:** Meta-regressions of the associations of patient characteristics with developmental quotient/intelligence quotient

	Number of studies	Number of patients	Slope (SE)	95% CI	τ^2^	*P* value
Duration of parenteral nutrition dependency in weeks	17	402	0.1 (0.1)	−0.1 to 0.1	65.5	0.347
Age at time of cognitive assessment in years	25	749	0.5 (0.7)	−0.8 to 1.8	95.0	0.527
Gestational age in weeks	23	636	1.1 (0.4)	0.4 to 1.8	43.9	0.041
Duration of hospital stay in weeks	21	668	−0.9 (0.2)	−1.3 to −0.5	51.6	0.014
Number of surgeries	13	367	−1.7 (0.7)	−3.1 to −0.3	30.7	0.033

Higher gestational age was associated with higher DQ/IQ, whereas longer hospital stay and higher number of surgeries were associated with lower DQ/IQ. Example: when a patient is hospitalized for 10 weeks longer, the patient's DQ/IQ is 9 points lower (with a slope of −0.9). CI = confidence interval; DQ = developmental quotient; IQ = intelligence quotient; SE = standard error; τ^2^ = tau-squared (represents the absolute value of true between-study variance, reflects heterogeneity).

## DISCUSSION

In this systematic review and meta-analysis, including 30 studies, we found that children with IF and surgically treated NEC have lower overall DQ/IQ and higher percentages of SDD compared with the general population. This was seen to a much lesser extent in children with abdominal wall defects and midgut malformations. Early, hospital admission-related factors but not duration of PN dependency, were predictive of developmental outcome.

There was a wide variation in mean DQ/IQ (72–102.3) and percentage of SDD (1%–51%) between studies; also within the same disease groups. Extent of disease may explain the variation. For example, in one of the studies, children with complex gastroschisis (accompanied by intestinal atresia, necrosis, perforation, and/or volvulus) had worse outcomes compared with simple gastroschisis patients; and complex gastroschisis patients are also the ones more likely to develop IF (19). Moreover, there may be underrepresentation of the actual clinical population, as in 10 studies, children with comorbidities, such as intraventricular haemorrhage, bronchopulmonary disease, and congenital syndromes were excluded (20,23–25,27,28,34,35,37,38), even though these are comorbidities that children with IF are often known with. Another explanation for variation in outcomes may be the variation in tools used to assess cognitive functioning, although these are all standardized and validated tools with the same mean and SD.

In the meta-regressions, risk factors for having lower DQ/IQ were shown to be lower gestational age, longer length of hospital, stay and higher number of surgical procedures.

A large part of the IF population is born preterm. Exponential brain growth occurs during fetal and infant maturation. A disruption of the organization of the brain of the neonate born prematurely can affect subsequent cognitive development (49). In several studies, preterm born children are found to have worse neurodevelopmental outcomes compared with term born children (50–52). In case-control studies included in the current meta-analysis, surgical NEC patients and gastroschisis patients had significantly lower DQ/IQ than gestational age-matched controls, suggesting that the impaired cognitive outcomes cannot be fully attributed to prematurity (25–27,29,30,38,41). Other factors, such as underlying inflammation, present in NEC and gastroschisis, may explain the differences in cognitive development (53–56).

Length of hospital stay was found to be a predictor of overall intelligence. This was also reported in large studies concerning infants after noncardiac surgery (57) and cardiac surgery (58). When infants are hospitalized for a long period of time, this may impede exploratory play, and thus delay cognitive development. Possibly, length of hospital stay is a proxy for the severity of illness that could explain the cognitive impairment.

The finding that surgery impacted cognitive development is supported by studies showing lower DQ/IQ in other patient populations requiring major neonatal surgery (59,60). In our meta-analysis, the association between surgery and developmental outcome seemed to be explained by 1 outlier study (35) with a mean of 11 surgical procedures (Figure S5, Supplemental Digital Content 7), supporting that most likely multiple surgeries are associated with impaired outcome. It is unclear what aspect of surgery is linked to the developmental changes. The role of anesthetics is subject of debate. A randomized controlled trial comparing infants undergoing surgery receiving general anesthesia with those receiving awake-regional anesthesia found no difference in developmental outcome at 5 years old (61). In that study, however, a single short length of anesthesia for a minor surgical procedure was examined. In other retrospective studies, longer or repeated anesthesia exposures were found to be associated with learning disabilities or worse DQ (62,63). A combination of exposure to general anesthesia and other perioperative factors is thought to make children vulnerable for memory impairment and school problems (64). Cerebral perfusion, nutritional and metabolic changes, physiologic stress, pain, and inflammation may impact neurodevelopment (65). In addition, just as length of hospital stay, the number of surgeries may be a proxy for critical illness.

Our results showed that there was no association between age at assessment and developmental outcome. Most studies, however, included children up to 2 years old. Little is known about the cognitive abilities of older children in the different underlying disease groups. In general, when children become older, tasks get more complex and demanding and deficits may become more apparent as a result of growing into deficit.

We expected a longer duration of PN dependency to be associated with lower DQ/IQ as there is growing evidence that early nutrition (especially essential fatty acids, zinc, and iron) could have long-term influence on cognitive abilities (66). PN can differ in composition of macronutrients and micronutrients from enteral nutrition. Also, PN is given through a central venous line, which is often accompanied by recurrent infections and limited freedom of movement, affecting cognitive development (67–69). The expected association was not confirmed in the meta-regression, which is reassuring.

The risk factors from the univariable meta-regressions may interact with one another but because of the limited number of studies (<10) with data on all predictors together, we were not able to perform a multivariable meta-regression. There may be other predictors of cognition in children with IF that we could not include in the meta-regressions. For example, changes in gut microbiota, also seen in pediatric patients with IF (70), are thought to influence cognition (71). The role of having a central venous line and other disease-specific factors of IF remain unclear in this matter.

We present the first meta-analysis on cognitive outcomes in both pediatric patients with IF and patients at risk of IF with conditions affecting the small intestine. The review's main strengths are its adherence to a registered protocol and methodologic advantages. Our study has several limitations that need to be taken into account when interpreting the results. First, most studies were retrospective with small sample sizes and limited follow-up time. Also, only 2 studies on midgut malformations were found, and no studies concerning enteropathies or motility disorders. Second, pooling of observation data without access to individual patient data is a limitation of meta-analyses in general. Therefore, we could not separate patients with PN dependency at the time of cognitive assessment from patients without PN. Another issue concerning PN and IF is that cut-offs of PN duration used for the definition of IF often differed or were not provided. Third, we had to transform medians to means for several patient characteristics for the meta-analysis. This may have led to an overestimation or underestimation of DQ/IQ and PN dependency duration. The widespread confidence intervals of outcomes shows the heterogeneity and indicates that the pooled estimates of the current meta-analyses are less precise and should be interpreted with caution. We chose to include multiple measures for defining developmental outcome, which may explain the heterogeneity too.

Cognitive development is a child's evolving ability to think and understand. It is important to detect alterations in cognitive functioning in an early stage, to stimulate development as soon as possible. Often, only medical predictors are evaluated but we know that also psychological factors, such as parent-child attachment and emotional functioning are associated with cognitive development (72,73). Future research should focus on gaining more insight into both medical and psychological risk and protecting factors for developing intellectual disabilities in children with and at risk of IF in order to create prevention and remediation strategies.

## CONCLUSIONS

In conclusion, our systematic review and meta-analysis showed that in patients with conditions affecting the small intestine requiring PN, children with IF and surgical NEC have a higher risk of developing adverse cognitive outcomes. Those with a low gestational age, long hospitalization, and multiple surgical procedures are especially prone. As survival rates of children with IF are improving, the number of at-risk patients is increasing. Therefore, it is important to monitor cognitive development in this vulnerable patient population and explore avenues for prevention and remediation whenever possible.

## Supplementary Material

Supplemental Digital Content

## Supplementary Material

Supplemental Digital Content

## Supplementary Material

Supplemental Digital Content

## Supplementary Material

Supplemental Digital Content

## Supplementary Material

Supplemental Digital Content

## Supplementary Material

Supplemental Digital Content

## Supplementary Material

Supplemental Digital Content

## Supplementary Material

Supplemental Digital Content

## References

[1] Goulet O Ruemmele F. Causes and management of intestinal failure in children. *Gastroenterology* 2006; 130: (2 Suppl 1): S16–S28. 16473066 10.1053/j.gastro.2005.12.002

[2] Jones IH Hall NJ. Contemporary outcomes for infants with necrotizing enterocolitis-a systematic review. *J Pediatr* 2020; 220:86.e3–92 e3. 31982088 10.1016/j.jpeds.2019.11.011

[3] Hijkoop A Rietman AB Wijnen RMH. Omphalocele at school age: what do parents report? A call for long-term follow-up of complex omphalocele patients. *Early Hum Dev* 2019; 137:104830. 31374454 10.1016/j.earlhumdev.2019.104830

[4] Hijkoop A Rietman AB Wijnen RMH. Gastroschisis at school age: what do parents report? *Eur J Pediatr* 2019; 178:1405–1412. 31325028 10.1007/s00431-019-03417-5 PMC6694033

[5] Dalla Vecchia LK Grosfeld JL West KW. Intestinal atresia and stenosis: a 25-year experience with 277 cases. *Arch Surg* 1998; 133:490–496. 9605910 10.1001/archsurg.133.5.490

[6] Thapar N Saliakellis E Benninga MA. Paediatric intestinal pseudo-obstruction: evidence and consensus-based recommendations from an ESPGHAN-Led Expert Group. *J Pediatr Gastroenterol Nutr* 2018; 66:991–1019. 29570554 10.1097/MPG.0000000000001982

[7] Ruemmele FM Schmitz J Goulet O. Microvillous inclusion disease (microvillous atrophy). *Orphanet J Rare Dis* 2006; 1:22. 16800870 10.1186/1750-1172-1-22 PMC1523325

[8] Hukkinen M Merras-Salmio L Pakarinen MP. Health-related quality of life and neurodevelopmental outcomes among children with intestinal failure. *Semin Pediatr Surg* 2018; 27:273–279. 30342603 10.1053/j.sempedsurg.2018.07.004

[9] Moher D Liberati A Tetzlaff J. Preferred reporting items for systematic reviews and meta-analyses: the PRISMA statement. *J Clin Epidemiol* 2009; 62:1006–1012. 19631508 10.1016/j.jclinepi.2009.06.005

[10] Cheong JLY Olsen JE Lee KJ. Temporal trends in neurodevelopmental outcomes to 2 years after extremely preterm birth. *JAMA Pediatr* 2021; 175:1035–1042. 34279561 10.1001/jamapediatrics.2021.2052 PMC8290336

[11] Development and use of quality assessment tools. Available at: *https://www.nhlbi.nih.gov/health-topics/study-quality-assessment-tools*. Accessed June 01, 2021.

[12] Wan X Wang W Liu J. Estimating the sample mean and standard deviation from the sample size, median, range and/or interquartile range. *BMC Med Res Methodol* 2014; 14:135. 25524443 10.1186/1471-2288-14-135 PMC4383202

[13] Hozo SP Djulbegovic B Hozo I. Estimating the mean and variance from the median, range, and the size of a sample. *BMC Med Res Methodol* 2005; 5:13. 15840177 10.1186/1471-2288-5-13 PMC1097734

[14] Maulik PK Mascarenhas MN Mathers CD. Prevalence of intellectual disability: a meta-analysis of population-based studies. *Res Dev Disabil* 2011; 32:419–436. 21236634 10.1016/j.ridd.2010.12.018

[15] Higgins JP Thompson SG Deeks JJ. Measuring inconsistency in meta-analyses. *BMJ* 2003; 327:557–560. 12958120 10.1136/bmj.327.7414.557 PMC192859

[16] Egger M Davey Smith G Schneider M. Bias in meta-analysis detected by a simple, graphical test. *BMJ* 1997; 315:629–634. 9310563 10.1136/bmj.315.7109.629 PMC2127453

[17] Balduzzi S Rucker G Schwarzer G. How to perform a meta-analysis with R: a practical tutorial. *Evid Based Ment Health* 2019; 22:153–160. 31563865 10.1136/ebmental-2019-300117 PMC10231495

[18] Viechtbauer W. Conducting meta-analyses in R with the metafor package. *J Stat Softw* 2010; 36:1–48.

[19] Hijkoop A H IJ Wijnen RMH. Prenatal markers and longitudinal follow-up in simple and complex gastroschisis. *Arch Dis Child Fetal Neonatal Ed* 2018; 103:F126–F131. 28615305 10.1136/archdischild-2016-312417

[20] Sirichaipornsak S. Neurodevelopmental outcomes of children with gastroschisis at university hospital in northeast Thailand. *Asian Biomed* 2011; 5:61.

[21] Sung SI Lee NH Kim HH. The impact of surgical intervention on neurodevelopmental outcomes in very low birth weight infants: a Nationwide Cohort Study in Korea. *J Korean Med Sci* 2019; 34:e271. 31701701 10.3346/jkms.2019.34.e271 PMC6838604

[22] Kuik SJ den Heijer AE Mebius MJ. Time to full enteral feeding after necrotizing enterocolitis in preterm-born children is related to neurodevelopment at 2-3 years of age. *Early Hum Dev* 2020; 147:105091. 32492527 10.1016/j.earlhumdev.2020.105091

[23] Hijkoop A Peters NCJ Lechner RL. Omphalocele: from diagnosis to growth and development at 2 years of age. *Arch Dis Child Fetal Neonatal Ed* 2019; 104:F18–F23. 29563149 10.1136/archdischild-2017-314700

[24] Adesanya OA O'Shea TM Turner CS. Intestinal perforation in very low birth weight infants: growth and neurodevelopment at 1 year of age. *J Perinatol* 2005; 25:583–589. 16034475 10.1038/sj.jp.7211360

[25] Allendorf A Dewitz R Weber J. Necrotizing enterocolitis as a prognostic factor for the neurodevelopmental outcome of preterm infants - match control study after 2years. *J Pediatr Surg* 2018; 53:1573–1577. 29409620 10.1016/j.jpedsurg.2018.01.006

[26] Hintz SR Kendrick DE Stoll BJ. NICHD Neonatal Research Network. Neurodevelopmental and growth outcomes of extremely low birth weight infants after necrotizing enterocolitis. *Pediatrics* 2005; 115:696–703. 15741374 10.1542/peds.2004-0569

[27] Lap CC Bolhuis SW Van Braeckel KN. Functional outcome at school age of children born with gastroschisis. *Early Hum Dev* 2017; 106–107:47–52. 10.1016/j.earlhumdev.2017.01.005 28189001

[28] O’Connor MJ Ralston CW Ament ME. Intellectual and perceptual-motor performance of children receiving prolonged home total parenteral nutrition. *Pediatrics* 1988; 81:231–236. 3124075

[29] Shah TA Meinzen-Derr J Gratton T. Hospital and neurodevelopmental outcomes of extremely low-birth-weight infants with necrotizing enterocolitis and spontaneous intestinal perforation. *J Perinatol* 2012; 32:552–558. 22157625 10.1038/jp.2011.176 PMC3496418

[30] Wadhawan R Oh W Hintz SR. NICHD Neonatal Research Network. Neurodevelopmental outcomes of extremely low birth weight infants with spontaneous intestinal perforation or surgical necrotizing enterocolitis. *J Perinatol* 2014; 34:64–70. 24135709 10.1038/jp.2013.128 PMC3877158

[31] Gold A Danguecan A Belza C. Neurocognitive functioning in early school-age children with intestinal failure. *J Pediatr Gastroenterol Nutr* 2020; 70:225–231. 31978022 10.1097/MPG.0000000000002500

[32] Zozaya C Shah J Pierro A. Canadian Neonatal Network (CNN) and the Canadian Neonatal Follow-Up Network (CNFUN) Investigators. Neurodevelopmental and growth outcomes of extremely preterm infants with necrotizing enterocolitis or spontaneous intestinal perforation. *J Pediatr Surg* 2021; 56:309–316. 32553453 10.1016/j.jpedsurg.2020.05.013

[33] Burnett AC Gunn JK Hutchinson EA. Cognition and behaviour in children with congenital abdominal wall defects. *Early Hum Dev* 2018; 116:47–52. 29136542 10.1016/j.earlhumdev.2017.11.002

[34] Bevilacqua F Rava L Valfre L. Factors affecting short-term neurodevelopmental outcome in children operated on for major congenital anomalies. *J Pediatr Surg* 2015; 50:1125–1129. 25783326 10.1016/j.jpedsurg.2014.12.015

[35] Chesley PM Sanchez SE Melzer L. Neurodevelopmental and cognitive outcomes in children with intestinal failure. *J Pediatr Gastroenterol Nutr* 2016; 63:41–45. 26655946 10.1097/MPG.0000000000001067 PMC4902780

[36] Danzer E Gerdes M D’Agostino JA. Prospective, interdisciplinary follow-up of children with prenatally diagnosed giant omphalocele: short-term neurodevelopmental outcome. *J Pediatr Surg* 2010; 45:718–723. 20385277 10.1016/j.jpedsurg.2009.09.004

[37] Elsinga RM Roze E Van Braeckel KN. Motor and cognitive outcome at school age of children with surgically treated intestinal obstructions in the neonatal period. *Early Hum Dev* 2013; 89:181–185. 23084574 10.1016/j.earlhumdev.2012.09.014

[38] Fullerton BS Hong CR Velazco CS. Severe neurodevelopmental disability and healthcare needs among survivors of medical and surgical necrotizing enterocolitis: a prospective cohort study. *J Pediatr Surg* 2018; 53:101–107. 10.1016/j.jpedsurg.2017.10.029 29079317

[39] Ginn-Pease ME King DR Tarnowski KJ. Psychosocial adjustment and physical growth in children with imperforate anus or abdominal wall defects. *J Pediatr Surg* 1991; 26:1129–1135. 1834823 10.1016/0022-3468(91)90688-p

[40] Harris EL Hart SJ Minutillo C. The long-term neurodevelopmental and psychological outcomes of gastroschisis: A cohort study. *J Pediatr Surg* 2016; 51:549–553. 26490011 10.1016/j.jpedsurg.2015.08.062

[41] Martin CR Dammann O Allred EN. Neurodevelopment of extremely preterm infants who had necrotizing enterocolitis with or without late bacteremia. *J Pediatr* 2010; 157:751.e1–756.e1. 20598317 10.1016/j.jpeds.2010.05.042 PMC2952050

[42] So S Patterson C Gold A. Neurodevelopmental outcomes of infants with intestinal failure at 12 and 26 months corrected age. *Early Hum Dev* 2019; 130:38–43. 30660017 10.1016/j.earlhumdev.2018.12.020

[43] Sorrell M Moreira A Green K. Favorable outcomes of preterm infants with parenteral nutrition-associated liver disease treated with intravenous fish oil-based lipid emulsion. *J Pediatr Gastroenterol Nutr* 2017; 64:783–788. 28437326 10.1097/MPG.0000000000001397

[44] South AP Marshall DD Bose CL. Growth and neurodevelopment at 16 to 24 months of age for infants born with gastroschisis. *J Perinatol* 2008; 28:702–706. 18615088 10.1038/jp.2008.71

[45] Ta BD Roze E van Braeckel KN. Long-term neurodevelopmental impairment in neonates surgically treated for necrotizing enterocolitis: enterostomy associated with a worse outcome. *Eur J Pediatr Surg* 2011; 21:58–64. 21157690 10.1055/s-0030-1267976

[46] van Manen M Hendson L Wiley M. Early childhood outcomes of infants born with gastroschisis. *J Pediatr Surg* 2013; 48:1682–1687. 23932607 10.1016/j.jpedsurg.2013.01.021

[47] Gunnar RJ Kanerva K Salmi S. Neonatal intestinal failure is independently associated with impaired cognitive development later in childhood. *J Pediatr Gastroenterol Nutr* 2020; 70:64–71. 31651669 10.1097/MPG.0000000000002529

[48] Humberg A Spiegler J Fortmann MI. German Neonatal Network (GNN). Surgical necrotizing enterocolitis but not spontaneous intestinal perforation is associated with adverse neurological outcome at school age. *Sci Rep* 2020; 10:2373. 32047169 10.1038/s41598-020-58761-6 PMC7012917

[49] Aylward GP. Neurodevelopmental outcomes of infants born prematurely. *J Dev Behav Pediatr* 2014; 35:394–407. 25007063 10.1097/01.DBP.0000452240.39511.d4

[50] Woythaler MA McCormick MC Smith VC. Late preterm infants have worse 24-month neurodevelopmental outcomes than term infants. *Pediatrics* 2011; 127:e622–e629. 21321024 10.1542/peds.2009-3598

[51] Larroque B Ancel PY Marret S. Neurodevelopmental disabilities and special care of 5-year-old children born before 33 weeks of gestation (the EPIPAGE study): a longitudinal cohort study. *Lancet* 2008; 371:813–820. 18328928 10.1016/S0140-6736(08)60380-3

[52] Serenius F Kallen K Blennow M. Neurodevelopmental outcome in extremely preterm infants at 2.5 years after active perinatal care in Sweden. *JAMA* 2013; 309:1810–1820. 23632725 10.1001/jama.2013.3786

[53] Lee SE West KP Jr Cole RN. General intelligence is associated with subclinical inflammation in Nepalese children: A population-based plasma proteomics study. *Brain Behav Immun* 2016; 56:253–263. 27039242 10.1016/j.bbi.2016.03.023 PMC4929134

[54] Kyriklaki A Margetaki K Kampouri M. Association between high levels of inflammatory markers and cognitive outcomes at 4years of age: the Rhea mother-child cohort study, Crete, Greece. *Cytokine* 2019; 117:1–7. 30772773 10.1016/j.cyto.2019.01.010 PMC8801160

[55] Moschopoulos C Kratimenos P Koutroulis I. The neurodevelopmental perspective of surgical necrotizing enterocolitis: the role of the gut-brain axis. *Mediators Inflamm* 2018; 2018:7456857. 29686534 10.1155/2018/7456857 PMC5866871

[56] Owaki T Imai K Miki R. Multiple cytokine analysis in gastroschisis: association with adverse outcomes including fetal brain damage. *Cytokine* 2021; 138:155406. 33341000 10.1016/j.cyto.2020.155406

[57] Batta V Rao S Wagh D. Early neurodevelopmental outcomes of congenital gastrointestinal surgical conditions: a single-centre retrospective study. *BMJ Paediatr Open* 2020; 4:e000736. 10.1136/bmjpo-2020-000736 PMC7422631 32821861

[58] Newburger JW Wypij D Bellinger DC. Length of stay after infant heart surgery is related to cognitive outcome at age 8 years. *J Pediatr* 2003; 143:67–73. 12915826 10.1016/S0022-3476(03)00183-5

[59] Meuwly E Feldmann M Knirsch W. Postoperative brain volumes are associated with one-year neurodevelopmental outcome in children with severe congenital heart disease. *Sci Rep* 2019; 9:10885. 31350426 10.1038/s41598-019-47328-9 PMC6659678

[60] Danzer E Hoffman C D’Agostino JA. Neurodevelopmental outcomes at 5years of age in congenital diaphragmatic hernia. *J Pediatr Surg* 2017; 52:437–443. 27622588 10.1016/j.jpedsurg.2016.08.008

[61] McCann ME de Graaff JC Dorris L. Neurodevelopmental outcome at 5 years of age after general anaesthesia or awake-regional anaesthesia in infancy (GAS): an international, multicentre, randomised, controlled equivalence trial. *Lancet* 2019; 393:664–677. 30782342 10.1016/S0140-6736(18)32485-1 PMC6500739

[62] Flick RP Katusic SK Colligan RC. Cognitive and behavioral outcomes after early exposure to anesthesia and surgery. *Pediatrics* 2011; 128:e1053–e1061. 21969289 10.1542/peds.2011-0351 PMC3307194

[63] Glatz P Sandin RH Pedersen NL. Association of anesthesia and surgery during childhood with long-term academic performance. *JAMA Pediatr* 2017; 171:e163470. 27820621 10.1001/jamapediatrics.2016.3470

[64] Schiller R H IJ Hoskote A. Memory deficits following neonatal critical illness: a common neurodevelopmental pathway. *Lancet Child Adolesc Health* 2018; 2:281–289. 30169299 10.1016/S2352-4642(17)30180-3

[65] Davidson AJ Vutskits L. Anesthesia in childhood and neurodevelopmental outcome. *Anesthesiology* 2020; 133:967–969. 32936864 10.1097/ALN.0000000000003551

[66] Anjos T Altmae S Emmett P. NUTRIMENTHE Research Group. Nutrition and neurodevelopment in children: focus on NUTRIMENTHE project. *Eur J Nutr* 2013; 52:1825–1842. 23884402 10.1007/s00394-013-0560-4

[67] Khalid Ijaz M R Rubino J. Impact of infectious diseases on cognitive development in childhood and beyond: potential mitigational role of hygiene. *Open Infect Dis J* 2012; 6:65–70.

[68] Benton D. ILSI Europe a.i.s.b.l. The influence of children's diet on their cognition and behavior. *Eur J Nutr* 2008; 47 Suppl 3:25–37. 18683027 10.1007/s00394-008-3003-x

[69] Grantham-McGregor S Baker-Henningham H. Review of the evidence linking protein and energy to mental development. *Public Health Nutr* 2005; 8:1191–1201. 16277829 10.1079/phn2005805

[70] Neelis E de Koning B Rings E. The gut microbiome in patients with intestinal failure: current evidence and implications for clinical practice. *JPEN J Parenter Enteral Nutr* 2019; 43:194–205. 30070709 10.1002/jpen.1423

[71] Mohajeri MH La Fata G Steinert RE. Relationship between the gut microbiome and brain function. *Nutr Rev* 2018; 76:481–496. 29701810 10.1093/nutrit/nuy009

[72] Stievenart M Roskam I Meunier JC. The reciprocal relation between children's attachment representations and their cognitive ability. *Int J Behav Dev* 2011; 35:58–66.

[73] Simeonsson RJ Rosenthal SL. Psychological and developmental assessment: children with disabilities and chronic conditions. New York: The Guilford Press; 2001.

