# Supplementary material for: Cognitive Outcomes in Children With Conditions Affecting the Small Intestine: A Systematic Review and Meta-analysis
Source: J Pediatr Gastroenterol Nutr. 2021 Dec 15;74(3):368–76. doi: 10.1097/MPG.0000000000003368 (PMC8860224; doi:10.1097/MPG.0000000000003368)
Supplement: Supplemental Digital Content [file jpga-74-368-s003.docx]

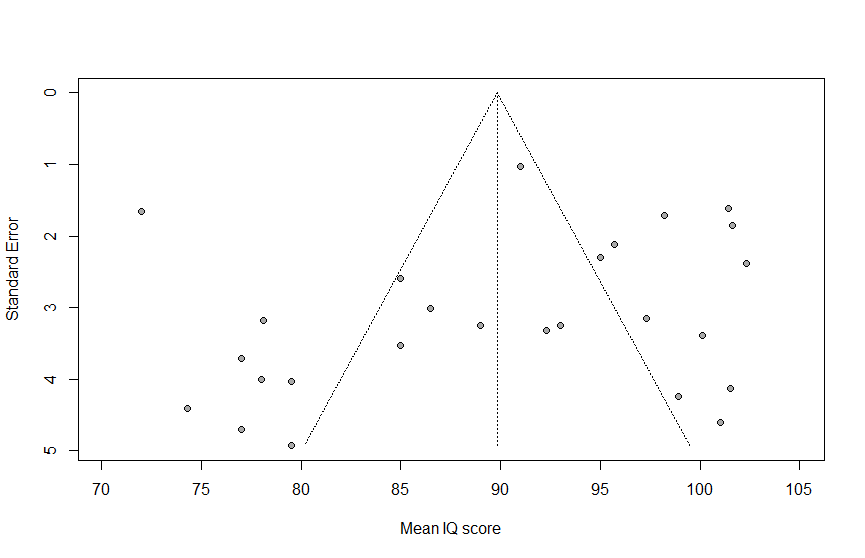


**Figure S1**. Funnel plot of the meta-analysis of studies reporting on DQ/IQ. Each plotted dot represents the mean DQ/IQ and standard error of a single study. The triangle represents the region in which 95% of the data points would lie in the absence of publication bias. The vertical dashed line represents the pooled overall mean DQ/IQ found in the meta-analysis. The funnel plot shows asymmetry indicating publication bias.

DQ: developmental quotient, IQ: intelligence quotient.

Mean DQ/IQ
