# Supplementary material for: Cognitive Outcomes in Children With Conditions Affecting the Small Intestine: A Systematic Review and Meta-analysis
Source: J Pediatr Gastroenterol Nutr. 2021 Dec 15;74(3):368–76. doi: 10.1097/MPG.0000000000003368 (PMC8860224; doi:10.1097/MPG.0000000000003368)
Supplement: Supplemental Digital Content [file jpga-74-368-s004.docx]

**Figure S2**. Funnel plot of the meta-analysis of studies reporting on percentage of patients with severe developmental delay/disability (developmental/intelligence quotient <70). Each plotted dot represents the percentage of patients with severe developmental delay/disability and standard error of a single study. The triangle represents the region in which 95% of the data points would lie in the absence of publication bias. The vertical dashed line represents the pooled overall percentage with severe developmental delay/disability found in the meta-analysis. The funnel plot shows asymmetry indicating publication bias.


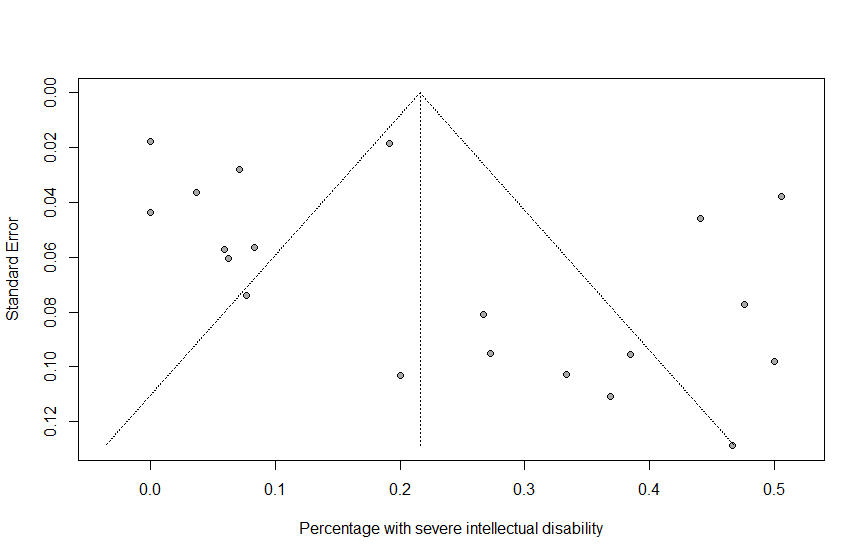


0 10 20 30 40 50

Percentage with severe developmental delay/disability
