# Supplementary material for: Cognitive Outcomes in Children With Conditions Affecting the Small Intestine: A Systematic Review and Meta-analysis
Source: J Pediatr Gastroenterol Nutr. 2021 Dec 15;74(3):368–76. doi: 10.1097/MPG.0000000000003368 (PMC8860224; doi:10.1097/MPG.0000000000003368)
Supplement: Supplemental Digital Content [file jpga-74-368-s006.docx]

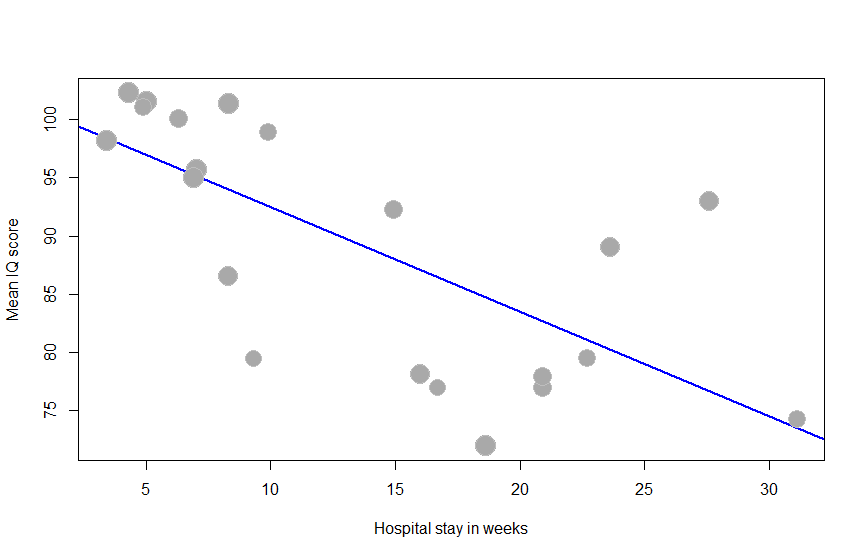


**Figure S4**. Scatterplot of the meta-regression analyzing the association between hospital stay in weeks and mean DQ/IQ. Each plotted dot represents the mean hospital stay in weeks and corresponding mean DQ/IQ of a single study. The decreasing regression line with negative slope indicates that a longer hospital stay in weeks is associated with lower mean DQ/IQ (*p* = 0.014).

DQ: developmental quotient, IQ: intelligence quotient.

Hospital stay in weeks

Mean DQ/IQ
