# Supplementary material for: Cognitive Outcomes in Children With Conditions Affecting the Small Intestine: A Systematic Review and Meta-analysis
Source: J Pediatr Gastroenterol Nutr. 2021 Dec 15;74(3):368–76. doi: 10.1097/MPG.0000000000003368 (PMC8860224; doi:10.1097/MPG.0000000000003368)
Supplement: Supplemental Digital Content [file jpga-74-368-s008.docx]

**File S1.** Search strategy in multiple electronic databases

**Embase – 3733 refs**

('intestinal failure'/de OR 'enteropathy'/de OR 'ileum dysfunction'/de OR 'intestinal dysmotility'/de OR 'malabsorption'/de OR 'intestine villus atrophy'/de OR 'short bowel syndrome'/de OR 'neurogenic bowel'/de OR 'gastroschisis'/de OR 'parenteral nutrition'/exp OR 'necrotizing enterocolitis'/de OR (((necrot*) NEAR/3 (enterocolit*)) OR ((parenteral* OR intravenous) NEAR/3 (nutrit* OR feed* OR hyperaliment* OR aliment*)) OR enteropath* OR gastroenteropath* OR gastro-enteropath* OR malabsorp* OR ileus* OR volvulus* OR gastroschis* OR ((intestin* OR gastrointest* OR duoden* OR ileum* OR jejun* OR ileal* OR microvillus* OR villous* OR microvillous*) NEAR/3 (syndrom* OR dysfunction* OR atrophy OR proteinlos* OR protein-los* OR atresia* OR anomal* OR failur* OR ischem* OR ischaem* OR necros* OR obstruct* OR occlus* OR pseudoobstruct* OR intussuscept* OR perforat* OR dysmotil* OR motil* OR hypermotil*)) OR ((bowel* OR short-gut*) NEAR/3 (failur* OR dysmotil* OR motil* OR hypermotil* OR neurogen* OR ischem* OR ischaem* OR necros* OR obstruct* OR occlus* OR pseudoobstruct* OR intussuscept* OR perforat* OR atresia* OR anomal* OR syndrom* OR short*))):ab,ti) **AND** ('cognition'/exp OR 'cognition assessment'/exp OR 'processing speed'/de OR 'language development'/de OR 'language processing'/de OR (cognit* OR neurocognit* OR metacognit* OR memor* OR neurodevelop* OR intellig* OR intellect* OR IQ OR EQ OR ((execut*) NEAR/3 (funct*)) OR visual-spatial OR visuospatial OR bayley OR Wechsler OR verbal* OR attention OR WPPSI OR WISC OR BSID OR ((processing) NEAR/3 (speed* OR velocit* OR rate* OR time*)) OR ((process* OR develop*) NEAR/3 (language*))):ab,ti) **AND** (child/exp OR adolescent/exp OR adolescence/exp OR 'child behavior'/de OR 'child parent relation'/de OR pediatrics/exp OR childhood/exp OR 'child nutrition'/de OR 'infant nutrition'/exp OR 'child welfare'/de OR 'child abuse'/de OR 'child advocacy'/de OR 'child development'/de OR 'child growth'/de OR 'child health'/de OR 'child health care'/exp OR 'child care'/exp OR 'childhood disease'/exp OR 'child death'/de OR 'child psychiatry'/de OR 'child psychology'/de OR 'pediatric ward'/de OR 'pediatric hospital'/de OR 'pediatric anesthesia'/de OR 'pediatric intensive care unit'/de OR 'neonatal intensive care unit'/de OR 'prematurity'/de OR (adolescen* OR preadolescen* OR infan* OR newborn* OR (new NEXT/1 born*) OR baby OR babies OR neonat* OR prematur* OR pre-matur* OR child* OR kid OR kids OR toddler* OR teen* OR boy* OR girl* OR minors OR underag* OR (under NEXT/1 (age* OR aging OR ageing)) OR juvenil* OR youth* OR kindergar* OR puber* OR pubescen* OR prepubescen* OR prepubert* OR pediatric* OR paediatric* OR school* OR preschool* OR highschool* OR suckling* OR PICU OR NICU OR PICUs OR NICUs):ab,ti) ***NOT ([animals]/lim NOT [humans]/lim)* NOT ([Conference Abstract]/lim)**

**Medline – 2053 refs**

(Malabsorption Syndromes/ OR Short Bowel Syndrome/ OR Neurogenic Bowel/ OR Gastroschisis/ OR exp Parenteral Nutrition/ OR Enterocolitis, Necrotizing/ OR (((necrot*) ADJ3 (enterocolit*)) OR ((parenteral* OR intravenous) ADJ3 (nutrit* OR feed* OR hyperaliment* OR aliment*)) OR enteropath* OR gastroenteropath* OR gastro-enteropath* OR malabsorp* OR ileus* OR volvulus* OR gastroschis* OR ((intestin* OR gastrointest* OR duoden* OR ileum* OR jejun* OR ileal* OR microvillus* OR villous* OR microvillous*) ADJ3 (syndrom* OR dysfunction* OR atrophy OR proteinlos* OR protein-los* OR atresia* OR anomal* OR failur* OR ischem* OR ischaem* OR necros* OR obstruct* OR occlus* OR pseudoobstruct* OR intussuscept* OR perforat* OR dysmotil* OR motil* OR hypermotil*)) OR ((bowel* OR short-gut*) ADJ3 (failur* OR dysmotil* OR motil* OR hypermotil* OR neurogen* OR ischem* OR ischaem* OR necros* OR obstruct* OR occlus* OR pseudoobstruct* OR intussuscept* OR perforat* OR atresia* OR anomal* OR syndrom* OR short*))).ab,ti.) **AND** (exp Cognition/ OR exp Intelligence Tests/ OR exp Language Development/ OR (cognit* OR neurocognit* OR metacognit* OR memor* OR neurodevelop* OR intellig* OR intellect* OR IQ OR EQ OR ((execut*) ADJ3 (funct*)) OR visual-spatial OR visuospatial OR bayley OR Wechsler OR verbal* OR attention OR WPPSI OR WISC OR BSID OR ((processing) ADJ3 (speed* OR velocit* OR rate* OR time*)) OR ((process* OR develop*) ADJ3 (language*))).ab,ti.) **AND** (exp Child/ OR exp Infant/ OR exp Adolescent/ OR exp "Child Behavior"/ OR exp "Parent Child Relations"/ OR exp "Pediatrics"/ OR "Child Nutrition Sciences"/ OR "Infant nutritional physiological phenomena"/ OR exp "Child Welfare"/ OR "Child Development"/ OR exp "Child Health Services"/ OR exp "Child Care"/ OR "Child Rearing"/ OR exp "Child development Disorders, Pervasive"/ OR "Child Psychiatry"/ OR "Child Psychology"/ OR "Hospitals, Pediatric"/ OR exp "Intensive Care Units, Pediatric"/ OR (adolescen* OR infan* OR newborn* OR (new ADJ born*) OR baby OR babies OR neonat* OR prematur* OR pre-matur* OR child* OR kid OR kids OR toddler* OR teen* OR boy* OR girl* OR minors OR underag* OR (under ADJ1 (age* OR aging OR ageing)) OR juvenil* OR youth* OR kindergar* OR puber* OR pubescen* OR prepubescen* OR prepubert* OR pediatric* OR paediatric* OR school* OR preschool* OR highschool* OR suckling* OR PICU OR NICU OR PICUs OR NICUs).ab,ti.) ***NOT (exp animals/ NOT humans/)* NOT (news OR congres* OR abstract* OR book* OR chapter* OR dissertation abstract*).pt.**

**Cochrane (RCTs) – 331 refs**

((((necrot*) NEAR/3 (enterocolit*)) OR ((parenteral* OR intravenous) NEAR/3 (nutrit* OR feed* OR hyperaliment* OR aliment*)) OR enteropath* OR gastroenteropath* OR gastro-enteropath* OR malabsorp* OR ileus* OR volvulus* OR gastroschis* OR ((intestin* OR gastrointest* OR duoden* OR ileum* OR jejun* OR ileal* OR microvillus* OR villous* OR microvillous*) NEAR/3 (syndrom* OR dysfunction* OR atrophy OR proteinlos* OR protein-los* OR atresia* OR anomal* OR failur* OR ischem* OR ischaem* OR necros* OR obstruct* OR occlus* OR pseudoobstruct* OR intussuscept* OR perforat* OR dysmotil* OR motil* OR hypermotil*)) OR ((bowel* OR short-gut*) NEAR/3 (failur* OR dysmotil* OR motil* OR hypermotil* OR neurogen* OR ischem* OR ischaem* OR necros* OR obstruct* OR occlus* OR pseudoobstruct* OR intussuscept* OR perforat* OR atresia* OR anomal* OR syndrom* OR short*))):ab,ti) **AND** ((cognit* OR neurocognit* OR metacognit* OR memor* OR neurodevelop* OR intellig* OR intellect* OR IQ OR EQ OR ((execut*) NEAR/3 (funct*)) OR visual-spatial OR visuospatial OR bayley OR Wechsler OR verbal* OR attention OR WPPSI OR WISC OR BSID OR ((processing) NEAR/3 (speed* OR velocit* OR rate* OR time*)) OR ((process* OR develop*) NEAR/3 (language*))):ab,ti) **AND** ((adolescen* OR preadolescen* OR infan* OR newborn* OR (new NEXT/1 born*) OR baby OR babies OR neonat* OR prematur* OR pre-matur* OR child* OR kid OR kids OR toddler* OR teen* OR boy* OR girl* OR minors OR underag* OR (under NEXT/1 (age* OR aging OR ageing)) OR juvenil* OR youth* OR kindergar* OR puber* OR pubescen* OR prepubescen* OR prepubert* OR pediatric* OR paediatric* OR school* OR preschool* OR highschool* OR suckling* OR PICU OR NICU OR PICUs OR NICUs):ab,ti)

**Web of Science – 1922 refs**

TS=(((((necrot*) NEAR/2 (enterocolit*)) OR ((parenteral* OR intravenous) NEAR/2 (nutrit* OR feed* OR hyperaliment* OR aliment*)) OR enteropath* OR gastroenteropath* OR gastro-enteropath* OR malabsorp* OR ileus* OR volvulus* OR gastroschis* OR ((intestin* OR gastrointest* OR duoden* OR ileum* OR jejun* OR ileal* OR microvillus* OR villous* OR microvillous*) NEAR/2 (syndrom* OR dysfunction* OR atrophy OR proteinlos* OR protein-los* OR atresia* OR anomal* OR failur* OR ischem* OR ischaem* OR necros* OR obstruct* OR occlus* OR pseudoobstruct* OR intussuscept* OR perforat* OR dysmotil* OR motil* OR hypermotil*)) OR ((bowel* OR short-gut*) NEAR/2 (failur* OR dysmotil* OR motil* OR hypermotil* OR neurogen* OR ischem* OR ischaem* OR necros* OR obstruct* OR occlus* OR pseudoobstruct* OR intussuscept* OR perforat* OR atresia* OR anomal* OR syndrom* OR short*)))) **AND** ((cognit* OR neurocognit* OR metacognit* OR memor* OR neurodevelop* OR intellig* OR intellect* OR IQ OR EQ OR ((execut*) NEAR/2 (funct*)) OR visual-spatial OR visuospatial OR bayley OR Wechsler OR verbal* OR attention OR WPPSI OR WISC OR BSID OR ((processing) NEAR/2 (speed* OR velocit* OR rate* OR time*)) OR ((process* OR develop*) NEAR/2 (language*)))) **AND** ((adolescen* OR preadolescen* OR infan* OR newborn* OR (new NEAR/1 born*) OR baby OR babies OR neonat* OR prematur* OR pre-matur* OR child* OR kid OR kids OR toddler* OR teen* OR boy* OR girl* OR minors OR underag* OR (under NEAR/1 (age* OR aging OR ageing)) OR juvenil* OR youth* OR kindergar* OR puber* OR pubescen* OR prepubescen* OR prepubert* OR pediatric* OR paediatric* OR school* OR preschool* OR highschool* OR suckling* OR PICU OR NICU OR PICUs OR NICUs)) NOT ((animal* OR rat OR rats OR mouse OR mice OR murine OR dog OR dogs OR canine OR cat OR cats OR feline OR rabbit OR cow OR cows OR bovine OR rodent* OR sheep OR ovine OR pig OR swine OR porcine OR veterinar* OR chick* OR zebrafish* OR baboon* OR nonhuman* OR primate* OR cattle* OR goose OR geese OR duck OR macaque* OR avian* OR bird* OR fish*) NOT (human* OR patient* OR women OR woman OR men OR man))) AND DT=(Article OR Review)

**PsycINFO – 150 refs**

(Irritable Bowel Syndrome/ OR Gastrointestinal Disorders/ OR (((necrot*) ADJ3 (enterocolit*)) OR ((parenteral* OR intravenous) ADJ3 (nutrit* OR feed* OR hyperaliment* OR aliment*)) OR enteropath* OR gastroenteropath* OR gastro-enteropath* OR malabsorp* OR ileus* OR volvulus* OR gastroschis* OR ((intestin* OR gastrointest* OR duoden* OR ileum* OR jejun* OR ileal* OR microvillus* OR villous* OR microvillous*) ADJ3 (syndrom* OR dysfunction* OR atrophy OR proteinlos* OR protein-los* OR atresia* OR anomal* OR failur* OR ischem* OR ischaem* OR necros* OR obstruct* OR occlus* OR pseudoobstruct* OR intussuscept* OR perforat* OR dysmotil* OR motil* OR hypermotil*)) OR ((bowel* OR short-gut*) ADJ3 (failur* OR dysmotil* OR motil* OR hypermotil* OR neurogen* OR ischem* OR ischaem* OR necros* OR obstruct* OR occlus* OR pseudoobstruct* OR intussuscept* OR perforat* OR atresia* OR anomal* OR syndrom* OR short*))).ab,ti.) **AND** (Cognition/ OR exp Intelligence Measures/ OR Bayley Scales of Infant Development/ OR exp Language Development/ OR (cognit* OR neurocognit* OR metacognit* OR memor* OR neurodevelop* OR intellig* OR intellect* OR IQ OR EQ OR ((execut*) ADJ3 (funct*)) OR visual-spatial OR visuospatial OR bayley OR Wechsler OR verbal* OR attention OR WPPSI OR WISC OR BSID OR ((processing) ADJ3 (speed* OR velocit* OR rate* OR time*)) OR ((process* OR develop*) ADJ3 (language*))).ab,ti.) **AND** (exp Childhood Development/ OR exp Child Care/ OR exp Early Childhood Development/ OR exp Adolescent Development/ OR exp "Parent Child Relations"/ OR exp OR "Pediatrics"/ OR exp "Child Welfare"/ OR exp "Child Psychiatry"/ OR "Child Psychology"/ OR Child Psychopathology/ OR (adolescen* OR infan* OR newborn* OR (new ADJ born*) OR baby OR babies OR neonat* OR prematur* OR pre-matur* OR child* OR kid OR kids OR toddler* OR teen* OR boy* OR girl* OR minors OR underag* OR (under ADJ1 (age* OR aging OR ageing)) OR juvenil* OR youth* OR kindergar* OR puber* OR pubescen* OR prepubescen* OR prepubert* OR pediatric* OR paediatric* OR school* OR preschool* OR highschool* OR suckling* OR PICU OR NICU OR PICUs OR NICUs).ab,ti.) ***NOT (exp animals/ NOT humans/)* NOT (news OR congres* OR abstract* OR book* OR chapter* OR dissertation abstract*).pt.**

**Google Scholar – 200 refs**

"intestine|intestinal|gastrointestinal|gastrointestine syndrome|dysfunction|failure|ischemia|ischaemia|necrosis"|"bowel failure|syndrome|short|ischemia|ischaemia|necrosis" cognition|memory|intelligence|intellect child|paediatric|pediatric
